# Reproducible Generalized Thermal Sensation Triggered by Levodopa in a Patient With Parkinson’s Disease

**DOI:** 10.14740/jmc5357

**Published:** 2026-07-28

**Authors:** Krittisak Anuroj, Numfah Paovanit

**Affiliations:** a Department of Psychiatry, Faculty of Medicine, Srinakharinwirot University, Nakhon Nayok, Thailand

**Keywords:** Parkinson’s disease, Levodopa, Dopaminergic drug, Thermal sensation, Heat sensation, Sensory disturbance, Adverse drug reaction, Drug-induced symptoms

## Abstract

Parkinson’s disease (PD) is characterized by motor impairments and diverse nonmotor symptoms, including sensory disturbances. Although chronic peripheral neuropathy has been described in association with long-term levodopa therapy, thermal sensory disturbance acutely following levodopa administration has not been previously reported. Here we describe a 69-year-old woman with an 8-year history of PD who developed acute generalized thermal (heat) sensations—initially misinterpreted as somatization—following exposure to several levodopa-containing pharmacological regimens. The phenomenon was subsequently reproduced during supervised medication rechallenge, but did not occur during monotherapy or combination therapy with dopamine agonists (pramipexole, rotigotine, and bromocriptine) and selegiline. During rechallenge, no objective changes in core body temperature, vasomotor signs, or sensory deficits were observed despite reproduction of the symptoms. Laboratory evaluation of metabolic and vitamin profiles, together with brain magnetic resonance imaging, excluded alternative causes of sensory disturbance. Given the acute onset and generalized distribution of symptoms, a central sensory mechanism was inferred, and potential relevance of dopaminergic signaling was discussed. The described phenomenon may represent a rare or underrecognized adverse effect of levodopa. As a part of comprehensive follow-up on patient’s health and wellbeing after treatment for PD, careful attention to emergent unusual thermal phenomena can constitute good clinical practice.

## Introduction

Parkinson’s disease (PD) is a neurodegenerative disorder clinically characterized by bradykinesia, coarse resting tremor, characteristic gaits, and nonmotor symptoms such as autonomic instability, depression, and sleep disturbances [1], while sensory disturbances such as pain and paresthesia have also been described [2]. Treatment commonly involves dopaminergic drugs aimed to restore and preserve ambulatory functions. Levodopa, a dopamine precursor used in combination with either carbidopa or benserazide, is one of the most commonly prescribed first-line agents [3–5]. Despite clear clinical benefits, levodopa has been associated with adverse effects such as gastrointestinal disturbances, postural hypotension, and neuropsychiatric symptoms such as impulsivity, psychosis, and confusion. Relevant to somatosensory adverse effects, levodopa has been associated with chronic sensory neuropathy, partly attributed to hyperhomocysteinemia and methylmalonic acid accumulation [6–8], although neuropathy can also emerge as a feature of PD, independent of levodopa exposure [9–11]. Nevertheless, acute somatosensory disturbances, particularly thermal sensations, in relation to levodopa administrations have been rarely reported. Here, we describe a patient with PD who repeatedly developed acute generalized heat sensations following administration of different levodopa formulations, with the symptoms initially misconstrued as somatization.

## Case Report

This case report describes a 69-year-old woman with PD, without other medical comorbidities, who repeatedly developed generalized heat sensations after exposure to levodopa. The case report was approved by the Human Research Ethics Committee of Srinakharinwirot University (SWUEC-693016). The patient was treated in accordance with the Declaration of Helsinki.

The patient’s PD was diagnosed 8 years earlier at a different hospital. Per history, the initial clinical presentation that led to evaluation and PD diagnosis was rigidity, subjectively perceived by the patient as limb heaviness and difficulty inhaling fully. Tremor was absent, reportedly contributing to initial delays in seeking evaluation for PD. Subsequent positron emission tomography imaging demonstrated dopaminergic abnormalities consistent with PD, including bilateral parietotemporal hypometabolism and increased fluorodeoxyglucose uptake in the bilateral caudate and putamen. Pharmacological treatment was reportedly initiated, though specific medication and dose information are unavailable; the patient denied medication-related adverse effects at that time.

Five years prior, following relocation and a change in treating hospital, her regimen was switched to a levodopa-containing agent (levodopa–benserazide; dose history unavailable). This reportedly resulted in improvement of rigidity, but also led to partial worsening of her breathing discomfort and the emergence of a generalized heat sensation after each dose of levodopa.

Three years prior, owing to suboptimal control of her rigidity, increasingly impairing shuffling gaits, and consequent limitations in activities of daily living, she sought treatment at the current hospital. Her modified Hoehn and Yahr stage at that time was 4. Her medication regimen was changed to levodopa–carbidopa (100/25 mg, one tablet, three times daily) in combination with ropinirole (2 mg/day); however, the heat sensation persisted. Subsequent escalation to levodopa–carbidopa (250/25 mg, half a tablet, four times daily), with the addition of rasagiline (0.5 mg/day) and rotigotine (2 mg/day), resulted in improvement of rigidity but worsened the heat sensation to an intolerable level, leading to poor medication adherence. During this period, constipation also worsened and required treatment with macrogol, elobixibat, and intermittent saline enemas.

One year prior, the patient’s gait had further deteriorated, with marked difficulty initiating gait, maintaining balance, and characteristic *en bloc* turning. These, combined with the progressed rigidity, rendered her almost bedridden. Tremor remained absent. The patient remained fully conscious without apparent cognitive impairment or perceptual disturbance. The treating neurologist rated her modified Hoehn and Yahr stage as 5. Dopaminergic treatment was further intensified (rasagiline increased to 1 mg/day and rotigotine to 4 mg/day), without recognizing her incomplete and intermittent medication adherence.

Although the patient reported persistent heat sensations to the treating neurologist, the temporal relationship to medication exposure was not clearly communicated. The symptoms were interpreted by the neurologist, and simultaneously perceived by her caregivers, as somatization. These prompted psychiatric referral and, after suspicion of a medication-related event was raised during case review, hospitalization for diagnostic clarification and supervised medication rechallenge.

Aside from the patient’s anxiety related to the heat sensation, psychiatric examination was unremarkable. The spontaneous episodes of heat sensation were not accompanied by features suggestive of panic attacks. Likewise, no associated motor, sensory, autonomic, or consciousness disturbances suggestive of paroxysmal neurological events were observed. A comprehensive neurological examination performed prior to the rechallenge revealed no abnormalities in tactile, proprioceptive, thermal, or pain sensation, and no peripheral vasomotor abnormalities were observed. Laboratory investigation for common causes of sensory neuropathy associated with PD, including deficiencies of vitamins B1, B12, and folate, as well as hyperhomocysteinemia, was unremarkable. Screening for diabetes mellitus was also negative. Thyroid function tests, liver and renal function tests, electrolyte levels, erythrocyte sedimentation rate, C-reactive protein, creatine phosphokinase, iron study, and total vitamin D levels were within normal limits. Brain magnetic resonance imaging (MRI) was unremarkable, with no evidence of cerebrovascular disease, white matter lesion, space-occupying lesion, or significant cerebral atrophy.

Medications were reintroduced one by one, each with a 24-h washout period. No additional medications or non-pharmacological treatments were prescribed throughout the rechallenge period. At the time of rechallenge, the patient’s body weight was 38 kg (body mass index, 16.2 kg/m^2^).

(1) Reintroduction of levodopa–carbidopa reproduced a generalized heat sensation at a dose of ½ tablet (100/25 mg), with a latency of approximately 30 min to symptom onset, a duration of 2–3 h, and greater symptom intensity with dose titration up to 1 tablet per dose.

(2) Levodopa–benserazide similarly reproduced symptoms at a dose of ¼ tablet (200/50 mg), with a latency of approximately 30 min and duration of 2–3 h.

(3) Administration of an orodispersible levodopa–benserazide formulation (100/25 mg) at ¼ tablet also consistently reproduced symptoms, with a shorter latency to onset of approximately 15 min and a duration of approximately 2 h.

Throughout all rechallenge episodes, no objective changes in core body temperature, peripheral vasomotor signs, or cutaneous findings were observed. No additional sensory symptoms were reported. No concurrent delusions or other perceptual disturbances were present; insight and reality testing were preserved. There were no features suggestive of cognitive impairment or delirium.

Subsequent monotherapy trials with pramipexole were well tolerated without inducing heat sensation up to a dose of 0.625 mg (two and a half regular tablet, four times daily.) Addition of 5–10 mg/day of selegiline and 2.5–5 mg/day of bromocriptine to the pramipexole regimen did not provoke thermal symptoms. Another separate monotherapy trial with rotigotine transdermal patch was also tolerated without heat sensation up to 6 mg/day. The patient was ultimately maintained on pramipexole and selegiline, which partially improved her rigidity and allowed caregiver-supported ambulation. A summary of medications inducing and not inducing the heat sensation is presented in Table 1.

**Table 1:** Medication History and Rechallenge Findings Related to Heat Sensation

Medication regimens
Producing heat symptoms (per history)
(1) Levodopa–benserazide monotherapy (unspecified formulation and dose)
(2) Levodopa–carbidopa monotherapy (100/25 mg TID)
(3) Combined levodopa–carbidopa (100/25 mg TID) and ropinirole (2 mg OD)
(4) Combined levodopa–carbidopa (125/12.5 mg QID), rasagiline (0.5 mg OD), and rotigotine patch (2 mg OD)
Producing heat symptoms (rechallenged)
(1) Levodopa–carbidopa (50/12.5–100/25 mg; dose-dependent intensity)
(2) Levodopa–benserazide (regular tablet; 50/12.5 mg)
(3) Levodopa–benserazide (orodispersible tablet; 25/6.25 mg)
Not producing heat symptoms during medication trial
(1) Pramipexole monotherapy (0.25–0.625 mg QID)
(2) Combined pramipexole (0.25–0.625 mg QID) and selegiline (5–10 mg OD)
(3) Combined pramipexole (0.25–0.625 mg QID), selegiline (5–10 mg OD), and bromocriptine (1.25–2.5 mg BID)
(4) Rotigotine patch monotherapy (2–6 mg/day, OD)

## Discussion

To our knowledge, this is the first documented case of reproducible acute thermal sensory symptoms elicited by multiple levodopa-containing formulations in a patient with PD. While alternative causes of the thermal sensation have been considered, they were deemed less likely based on the highly reproducible clinical pattern associated with levodopa exposure, the absence of features suggestive of psychiatric or neurological paroxysmal conditions, unremarkable laboratory investigations for common causes of neuropathy in PD, and normal brain MRI findings.

Past misinterpretation of the patient’s symptoms as somatization likely reflected a convergence of factors, including incomplete delineation of the temporal relationship with dopaminergic therapy and the rarity of this adverse effect. In older Asians, such poorly localized sensory complaints are commonly reported in association with affective disorders, potentially biasing their interpretation [12, 13]. In addition, the use of indirect and circumstantial emotional language and substitution of somatic terms for emotional communication (somatothymia) in Thai cultural settings [14–16] could have further complicated clinical evaluation and increased vulnerability to premature attribution of such symptoms to psychological conditions.

Overall, the temporal relationship with levodopa exposure and consistent pattern during rechallenge strongly supported a levodopa-induced phenomenon. It could be speculated from the rarity of reports of thermal sensations directly elicited by levodopa, despite its widespread prescription [3–5], that additional predisposing biological factors may underlie symptom manifestation in our patient.

Given the generalized distribution of the patient’s sensory symptoms and the absence of length-dependent or focal patterns that could suggest peripheral neuropathies, a central mechanism of sensory processing may be speculated in our case. Somatosensory thalamic sensory relay depolarization is affected by dopaminergic D1-like and D2-like receptors [17]. Neurophysiological studies suggested that thalamocortical sensory gating, as reflected by short-latency afferent inhibition, may be disrupted in PD, but the effects of dopaminergic drugs on this gating process have not been systematically evaluated [18–22]. Dopamine may also affect sensory processing at striatal level; its modulatory role in sensory processing is discussed in relation to reward and threat processing, in which dopamine and acetylcholine interact and affect attentional shifting and responsiveness to salient sensory inputs [23–28]. Dopamine in the prefrontal circuits influences glutamatergic transmission, neuronal excitability, and plasticity, particularly via D1 and D3 receptors. This modulation affects cognitive control and top-down attention process, which can then be relevant to selective processing of sensory information [29–31]. Accordingly, an experimental study has demonstrated that dopaminergic agonists modulate prefrontal cortical gating of sensory input [29].

Conscious perception of thermal signals involves primary and secondary somatosensory cortices (S1/S2); however, direct modulatory influence of dopamine within these regions remains incompletely understood [32]. Dopamine has nevertheless been implicated in somatosensory cortical plasticity and response to sensory stimulation [33].

The insular cortex is also involved in thermal processing, with sensory-discriminative and affective-cognitive components mapped to posterior and anterior subregions, respectively [34]. The region’s relevance to interoception, the awareness of bodily signals, is also well noted [35]. Again, the contribution of dopaminergic modulation within insular cortex remains incompletely characterized.

Our literature review conducted in PubMed and Scopus noted only a few related reports on thermal phenomena in patients with PD. One case report described a PD patient with heat intolerance as a part of PD presentation whose thermal symptoms improved soon after administration of levodopa [36]. Another case described a patient with intraoral burning sensation triggered by levodopa after 6 weeks of treatment, and the symptoms resolved after switching regimen to dopamine agonist [37]. None of these bear direct resemblance to the phenomena described in our case.

Similarly, there have been limited experimental and observational studies directly examining thermal phenomena in PD. Higher thermal sensory thresholds have been observed among PD patients, a feature apparently unaffected by levodopa treatment but responsive to deep brain stimulation [38]. Another study evaluating heat and cold pain thresholds—a related but distinct construct—reported increased sensitivity to thermal pain in PD patients, although this feature likewise appeared unaffected by levodopa treatment [39]. In contrast, heightened subjective heat sensitivity, partly attributed to dysregulated thermoregulation, has also been described in PD [40], further complicating the relationship between objective sensory thresholds and patient’s lived thermal experience. In healthy volunteers, acute dopaminergic depletion has been shown to alter thermal detection thresholds [33], but extrapolation of these findings to patients with PD, whose dopaminergic depletions have been chronic, can be challenging.

Owing to the paucity of literature on this topic, it remains unclear whether the levodopa-related generalized thermal sensations observed in our case represent a genuinely rare adverse event or a phenomenon that has been underrecognized and underexplored. The evidence regarding the role of dopamine and dopaminergic agents in thermal sensory processing has also been scarce, limiting our mechanistic inferences. In addition to the paucity of reports on thermal symptoms attributed to levodopa, similar reports involving other dopaminergic agents, such as bupropion, psychostimulants, and other antiparkinsonian drugs, have likewise been absent. Nevertheless, the observation that symptoms were associated with levodopa but not with other dopaminergic drugs in our case (Table 1) raises the possibility that differential dopaminergic receptor involvement might have been relevant to thermal sensory processing mechanisms; these could have interacted with additional preexistent vulnerability. This hypothesis remains speculative and warrants further mechanistic investigation.

### Conclusion

We document a female patient with PD who developed a reproducible, generalized heat sensation attributable to multiple formulations of levodopa, in the absence of objective changes in body temperature, focal sensory signs, psychosis, or alternative explanatory medical and psychiatric conditions. Based on our literature review, this phenomenon has not been previously reported. The limited available literature provides relevant evidence regarding the role of dopaminergic transmission in centrally mediated sensory processing to support the mechanistic hypotheses discussed herein; interaction with additional patient-specific vulnerability was presumed. Our report highlights a potentially underrecognized phenomenon in patients with PD undergoing levodopa treatment and underscores the importance of careful phenomenological assessment and medication rechallenge as part of a holistic, patient-centered approach to atypical sensory complaints. Clinicians should remain attentive to reports of levodopa-associated thermal sensory disturbances. If such phenomena prove more common than currently appreciated, further systematic investigation to clarify their underlying mechanisms can be justified.

## Data Availability

Data sharing is not applicable to this article, as no datasets were generated or analyzed.

## Abbreviations

MRI: magnetic resonance imaging
PD: Parkinson’s disease
